# Physiological Synchrony in EEG, Electrodermal Activity and Heart Rate Detects Attentionally Relevant Events in Time

**DOI:** 10.3389/fnins.2020.575521

**Published:** 2020-12-03

**Authors:** Ivo V. Stuldreher, Nattapong Thammasan, Jan B. F. van Erp, Anne-Marie Brouwer

**Affiliations:** ^1^Perceptual and Cognitive Systems, Netherlands Organisation for Applied Scientific Research (TNO), Soesterberg, Netherlands; ^2^Human Media Interaction, Faculty of Electrical Engineering, Mathematics and Computer Science, University of Twente, Enschede, Netherlands

**Keywords:** physiological synchrony, inter-subject correlations, interpersonal physiology, EEG, electrodermal activity, heart rate, multimodal

## Abstract

Interpersonal physiological synchrony (PS), or the similarity of physiological signals between individuals over time, may be used to detect attentionally engaging moments in time. We here investigated whether PS in the electroencephalogram (EEG), electrodermal activity (EDA), heart rate and a multimodal metric signals the occurrence of attentionally relevant events in time in two groups of participants. Both groups were presented with the same auditory stimulus, but were instructed to attend either to the narrative of an audiobook (audiobook-attending: AA group) or to interspersed emotional sounds and beeps (stimulus-attending: SA group). We hypothesized that emotional sounds could be detected in both groups as they are expected to draw attention involuntarily, in a bottom-up fashion. Indeed, we found this to be the case for PS in EDA or the multimodal metric. Beeps, that are expected to be only relevant due to specific “top-down” attentional instructions, could indeed only be detected using PS among SA participants, for EDA, EEG and the multimodal metric. We further hypothesized that moments in the audiobook accompanied by high PS in either EEG, EDA, heart rate or the multimodal metric for AA participants would be rated as more engaging by an independent group of participants compared to moments corresponding to low PS. This hypothesis was not supported. Our results show that PS can support the detection of attentionally engaging events over time. Currently, the relation between PS and engagement is only established for well-defined, interspersed stimuli, whereas the relation between PS and a more abstract self-reported metric of engagement over time has not been established. As the relation between PS and engagement is dependent on event type and physiological measure, we suggest to choose a measure matching with the stimulus of interest. When the stimulus type is unknown, a multimodal metric is most robust.

## Introduction

Knowing what events in the external environment people attend to, and how their shared attentional engagement to events varies over time, can be useful in a range of settings, from evaluating educational or entertaining material, to real time adjustment of important instructions. Unlike explicit measures, such as questionnaires in which people are asked to specify their attentional engagement, physiological signals can provide continuous and implicit information on mental state (Zander and Kothe, 2011). However, the link between mental state and physiological measures [e.g., electroencephalography (EEG), electrodermal activity (EDA) or heart rate] is not straightforward (Brouwer et al., 2015). A popular approach to uncover the complex links between physiology and mental state is the use of supervised learning algorithms. These algorithms predict mental state based on a set of features extracted from physiological variables (Hamadicharef et al., 2009; Hussain et al., 2011; Fleureau et al., 2012; Liu et al., 2013; Aliakbaryhosseinabadi et al., 2017). A disadvantage of these types of analyses is the need for labeled training data, i.e., a set of physiological data that are labeled with a known value for the mental state of interest. Not only is it time consuming to obtain such a labeled dataset, it is also very difficult to determine the ‘ground truth’ mental state than can be used for data labeling (Brouwer et al., 2015). A second drawback of these supervised learning approaches is that classification is often limited to a small number of discrete states. Attentional, emotional or cognitive state, however, cannot realistically be represented by a small number of discrete states, but are naturally of more continuous nature (Zehetleitner et al., 2012; Rosenberg et al., 2013).

For monitoring attentional engagement, an approach that may be suited to circumvent both of the abovementioned problems is to monitor the physiological synchrony (PS) between individuals. PS is the degree to which physiological measures of multiple people uniformly change. Studies exploring PS in functional magnetic resonance imaging data have revealed strong voxel-wise inter-subject correlations across participants exposed to a common narrative stimulus (Hasson et al., 2004, 2010; Hanson et al., 2009). In the faster EEG signals, similar results were found (Dmochowski et al., 2012, 2014). The fast-changing EEG enabled the computation of a continuous measure of PS in time and suggested that moments of high PS corresponded with emotionally arousing scenes of the movie clips (Poulsen et al., 2017). For instance, high PS was found when scenes were viewed that involved the threat of a gun. Dmochowski et al. (2014) further showed that moment-to-moment variation in the PS predicted the general expressions of interest and attention of the public as indicated by number of tweets during a popular television series. Davidesco et al. (2019) found that PS over time indicated what specific information was retained by students in a lecture. Namely, PS was higher in lecture parts that provided answers for questions that students answered incorrectly in the pre-test and correctly in the delayed post-test than for questions where students’ answers did not change. The relationship between neural PS and attentional engagement was also found to be less complex than most traditional physiological metrics. Neural PS was found to be directly proportional to attentional engagement, as strong correlations were found between PS and performance on questionnaires reflective of paid attention (Cohen and Parra, 2016; Cohen et al., 2018; Stuldreher et al., 2020). This directly proportional relationship may thus be used to circumvent supervised learning approaches and the problems that come with such approaches, such as the dependency on labeled training data.

In the current work, we aim to employ the relation between PS and attentional engagement to detect the occurrence of attentionally relevant events in time. Rather than limiting the analyses to EEG, we also include PS measures of peripheral nervous system activity (EDA and heart rate), and quantify their comparative sensitivity of detecting relevant events. Up to recently, PS in peripheral physiological measures has been studied mainly as a metric of some form of affective connectedness between individuals (reviewed by Palumbo et al., 2017). Examples include peripheral PS in therapist-patient dyads as a measure of psychotherapy success (Koole et al., 2020), in couples in marital counseling as a measure of therapy outcome (Tourunen et al., 2019) and as measure of collaborative learning (Malmberg et al., 2019). Positive results found in these contexts may (partly) be driven by shared attentional engagement to external events, as connectedness between people may be strongly associated with mutual attentiveness (Tickle-Degnen and Rosenthal, 1990). Recently, it was found that PS in EDA and heart rate can indeed reflect shared attention toward narrative stimuli (Pérez et al., 2020; Stuldreher et al., 2020).

The advantage of peripheral physiological measures over EEG is that they can be recorded more easily and less obtrusively. In addition, EEG and peripheral measures may complement each other since they likely reflect different mental processes. EEG is, for example, sensitive to selective attention (Polich, 2007), whereas EDA and heart rate are sensitive to (emotional) arousal (Cacioppo et al., 2007; Boucsein, 2012).

As of yet, it is unknown whether PS in EEG, EDA and heart rate can be used to detect relevant moments in time. For EDA and heart rate, time-resolved dynamics of PS have not been investigated at all in the context of attentional engagement. For EEG, time-resolved dynamics have been explored (see for instance Dmochowski et al., 2014; Poulsen et al., 2017), but this has not been done systematically, using a-priori known cognitively or emotionally engaging stimuli for which detection performance can be evaluated. We here evaluate whether PS in EEG, EDA and heart rate can be used to detect cognitively or emotionally relevant moments in time. Our goal is not to compare detection performance directly between the different types of stimuli, but to evaluate PS for a range of events differing in terms of total duration, sound onset, mental processes addressed and more. Just as in real-world conditions, some event may capture attention in a bottom-up fashion, related to salience or emotional relevance, whereas others may only capture attention due to top-down mechanisms related to task instruction (Lang, 1995; Öhman et al., 2001; Schupp et al., 2003). We invited participants to come to our lab and listen to an audiobook that was interspersed with short auditory events, that we expected to induce emotional and cognitive load. We divided the participants in two equal-sized groups. Participants in the audiobook-attending group (AA) were instructed to focus their attention on the audiobook and ignore the interspersed stimuli. Participants in the stimulus-attending group (SA) were instructed to focus their attention on the interspersed stimuli and ignore the audiobook. In a previous paper on this experiment (Stuldreher et al., 2020), we showed that PS can be used to correctly classify a listener as being instructed to attend to the audiobook or to the sounds. In the current paper, we use PS among individuals in the same group to predict the occurrence of interspersed stimuli over time, for each of the three physiological measures. In addition, we investigated if the PS across AA participants was predictive of the occurrence of engaging moments in the book. We aimed to answer the following research questions:

Does PS in EEG and EDA, heart rate and a multimodal metric predict the occurrence of attentionally engaging moments in time? And does this depend on the attentional instruction, type of stimuli and physiological measure?

We expect that interspersed stimulus detection performance of PS measures depends on combinations of the attentional group (AA or SA), the interspersed stimulus type (emotional sounds or beeps) and the physiological measure (EEG and EDA, heart rate or the multimodal metric). We hypothesized the following;

(1) Attentional instruction and stimulus type: (a) for the SA group, detection performance based on PS is above chance for all interspersed stimuli. (b) For the AA group, detection performance based on PS is above chance for emotional sounds, since these attract attention through bottom-up mechanisms related to salience or emotional relevance (Lang, 1995; Öhman et al., 2001; Schupp et al., 2003) irrespective of task instruction. (c) For the AA group, detection performance based on PS is not above chance for beeps, as these are expected to mainly attract attention through top-down mechanisms related to task-instructions.

(2) Physiological measure and stimulus type (a) PS based on peripheral signals (EDA and heart rate) performs better on the detection of emotional sounds than on beeps, because they primarily reflect emotional state (Cacioppo et al., 2007; Boucsein, 2012). (b) PS based on EEG performs better on the detection of beeps than on the detection of emotional sounds, because they primarily reflect top-down selective attention or mental effort (Hogervorst et al., 2014).

(3) Combining physiological measures: combining the physiological measures into a single multimodal metric of PS would result in relatively high detection accuracies when disregarding the differences between stimulus types.

While for the SA group, the timing of short stimuli serve as “ground truth” relevant events to compare to the moments of high PS, we do not know *a priori* what constitutes relevant events or engaging moments in the audiobook. We therefore investigate ratings of post-hoc determined moments of high and low PS in the audiobook by an independent group of participants. We hypothesized that;

(4) Events in audiobook: moments of the audiobook that were associated with high PS in the AA group are rated as more engaging than moments of the audiobook that were associated with low PS.

## Materials and Methods

### Participants

Twenty-seven participants (17 female), between 18 and 48 years old, with an average of 31.6 years and a standard deviation of 9.8 years, were recruited through the institute’s participant pool. Before performing the study, approval was obtained from the TNO Institutional Review Board (IRB). The approval is registered under the reference 2018–70. Prior to the experiment all participants signed informed consent, in accordance with the Declaration of Helsinki. After signing, all participants were randomly assigned to either the AA group or the SA group. After the experiment they received a small monetary reward for their time and traveling costs. None of the participants indicated problems in hearing or attention. Data of one participant were discarded due to failed physiological recordings, resulting in two equal-sized groups.

### Materials

EEG, EDA, and electrocardiogram (ECG) were recorded at 1024 Hz using an ActiveTwo Mk II system (BioSemi, Amsterdam, Netherlands). EEG was recorded with 32 active Ag-AgCl electrodes, placed on the scalp according to the 10–20 system, together with a common mode sense active electrode and driven right leg passive electrode for referencing. The electrode impedance threshold was maintained below 20 kOhm. For EDA, two passive gelled Nihon Kohden electrodes were placed on the ventral side of the distal phalanges of the middle and index finger. For ECG, two active gelled Ag-AgCl electrodes were placed at the right clavicle and lowest floating left rib. EDA and heart rate were also recorded using wearable systems (Movisens EdaMove 4 and Wahoo Tickr, respectively). These data are discussed elsewhere (Borovac et al., 2020; Van Beers et al., 2020).

### Stimuli and Design

Participants performed the experiment one by one. Each participant was presented with the exact same audio file, composed of a 66 min audiobook (a Dutch thriller “Zure koekjes,” written by Corine Hartman) interspersed with other short, auditory stimuli. Half of the participants were asked to focus on the narrative of the audiobook and ignore all other stimuli or instructions (AA group); and half of the participants were asked to focus on the short, interspersed stimuli and perform accompanying tasks, and ignore the narrative (SA group). The auditory stimuli were 36 emotional sounds, 27 blocks of beeps that SA participants had to keep track of, and an auditory instruction to sing a song. The order of sounds and beeps was randomly determined but was identical for each participant. Inter-stimulus intervals varied between 35 and 55 s, with an average of 45 s and a standard deviation of 6.1 s. We selected these stimuli to evaluate PS for a broad range of events, differing in e.g., audio profile and expected effect on mental processes as a function of task instructions.

Emotional sounds were taken from the second version of the International Affective Digitized Sounds (IADS) (Bradley and Lang, 2007). The IADS is a collection of 6-s acoustic stimuli that have been normatively rated for valence (positive or negative affect), arousal and dominance. Examples of stimuli are the sound of a crying baby or a cheering sports crowd. We selected 12 neutral sounds (IADS number 246, 262, 373, 376, 382, 627, 698, 700, 708, 720, 723, 728), 12 pleasant sounds (110, 200, 201, 202, 311, 352, 353, 365, 366, 367, 415, 717) and 12 unpleasant sounds (115, 255, 260, 276, 277, 278, 279, 285, 286, 290, 292, 422) based on their normative ratings of valence and arousal. We expected these sounds to attract attention of all participants, even those instructed to ignore the interspersed sounds.

Beeps were presented in blocks of 30 s, with every 2 s a 100 ms high (1 kHz) or low (250 Hz) pitched beep. SA participants needed to separately count the number of high and low beeps presented in a block, as in (De Dieuleveult et al., 2018). This task was practiced with them beforehand. In total, 27 blocks of beeps were presented. We expected these sounds to only attract attention of participants clearly instructed to keep track of them.

Toward the end of the audiobook, the instruction was presented to sing a song aloud after a subsequent auditory countdown reached 0. This instruction had to be followed by the SA group and was expected to induce stress and a strong increase in EDA and heart rate (Brouwer and Hogervorst, 2014). For the current analyses, data following the onset of this stimulus were discarded, because some participants started singing before the counter reached 0. This prohibited analysis of the data in terms of mental processes due to confounding movement effects and artifacts in the data recording.

In total, we consider 3,800 s of data in further analyses, out of which 1,026 s involved concurrent presentation of the audiobook and interspersed stimuli.

### Analysis

#### Pre-processing

Data processing was done using MATLAB 2019a software (Mathworks, Natick, MA, United States). For EEG pre-processing we also used EEGLAB v14.1.2 for MATLAB (Delorme and Makeig, 2004). To remove potentials not reflecting sources of neural activity, but ocular or muscle-related artifacts, logistic infomax independent component analysis (ICA) (Bell and Sejnowski, 1995) was performed. EEG was first down sampled to 256 Hz and high-pass filtered at 1 Hz. This relatively high cut-off frequency has shown to work better for ICA compared to lower cut-off frequencies (Winkler et al., 2015). Data were then notch filtered at 50 Hz, using the standard FIR-filter implemented in EEGLAB function pop_eegfiltnew. ICA was performed and the Multiple Artifact Rejection Algorithm (MARA) (Winkler et al., 2011) was used to identify artifactual independent components, i.e., components not reflecting sources of neural activity, but ocular or muscle-related artifacts. These components were removed from re-referenced, but uncleaned data. In these data, samples whose squared amplitude magnitude exceeded the mean-squared amplitude of that channel by more than four standard deviations were marked as missing data (“NaN”) in an iterative way with four repetitions. By doing so, 0.82 % of data were marked as missing.

EDA was downsampled to 32 Hz. The fast changing phasic and slowly varying tonic components of the signal were extracted using Continuous Decomposition Analysis as implemented in the Ledalab toolbox for MATLAB (Benedek and Kaernbach, 2010). In the further analyses we use the phasic component of the signal as this component of the EDA signal is mainly related to responses to external stimuli.

ECG measurements were processed to acquire the inter-beat interval (IBI – inversely proportional to heart rate). After downsampling to 256 Hz, ECG was high-pass filtered at 0.5 Hz. Peaks were detected following Pan and Tompkins (1985). The IBI semi-time series was transformed into a timeseries by interpolating consecutive intervals and resampling at 32 Hz.

#### Computation of Inter-Subject Correlations as Measure of Physiological Synchrony

We computed PS by measuring the inter-subject correlations of the neurophysiological signals. For EEG, rather than treating the signals from the 32 channels separately, we evaluated the inter-subject correlations in the correlated components of the EEG (Dmochowski et al., 2012, 2014). The goal of the correlated component analysis is to find underlying neural sources that are maximally correlated between participants, based on linear combinations of electrodes. Components were extracted separately from the AA group and SA group. EEG data from each participant were projected on the component vectors. Participant-to-group inter-subject correlations were then computed as the sum of correlations in the first three component projections, following (Dmochowski et al., 2012, 2014; Cohen and Parra, 2016; Ki et al., 2016; Cohen et al., 2018). Even though we used fewer participants in each attentional group than earlier work on auditory PS (e.g., Cohen and Parra, 2016; Ki et al., 2016), scalp projections of the components were very similar to those obtained in these earlier works, and our EEG PS values were in a similar range of 0.01 to 0.04. For the computation of time-resolved inter-subject correlations, correlations were computed in running 5 s windows at 1 s increments.

Inter-subject correlations in EDA and IBI were computed following (Marci et al., 2007). We computed Pearson correlations over successive, running 15 s windows at 1 s increments as measure of time-resolved inter-subject correlations. Participant-to-group correlations were computed by averaging over all correlations with all other participants in a group.

#### Physiological Synchrony for the Detection of Interspersed Stimuli

**FIGURE 1:**
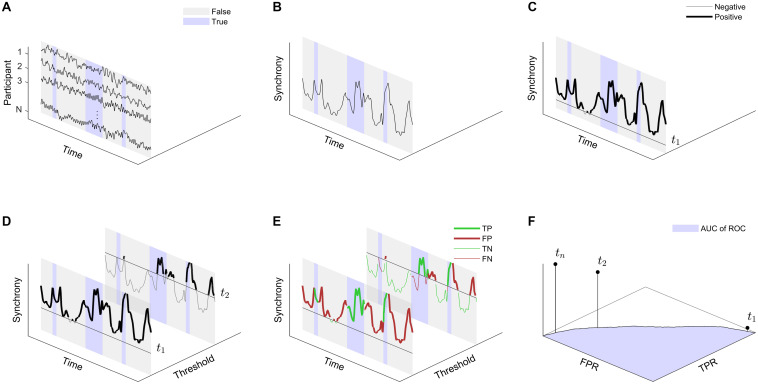
Illustration of the event detection paradigm. **(A)** Consider the physiological response traces recorded from *N* participants who were presented with the same external stimuli at the same time. The timestamps of the data recordings can be separated in moments where events were presented and where an event detection would thus be correct (True) and moments where no events where presented and where an event detection would thus be incorrect (False). **(B)** Rather than considering the raw physiological responses, the detection paradigm is based on the PS between the participants. **(C)** Now let us define a threshold *t*. The moments in time where the synchrony is higher than *t* are marked as an event (Positive) and the moments in time where the synchrony is lower than *t* are marked as a non-event (Negative). **(D)** Rather than using a single value for *t*, we consider a gradually changing threshold *t*_0_ to *t*_*n*_, so that at *t*_0_ all data are marked as Positive and at *t*_*n*_ all data are marked as Negative **(E)** For each iteration *t*_*i*_, we can now define the true positives (TP), false positives (FP), true negatives (TN), and false negatives (FN) and thus compute the true-positive rate (TPR) and false-positive rate (FPR). **(F)** Plotting the FPR versus the TPR – both as a function of *t* – results in the receiver operating curve (ROC). Detection performance is defined as the standard metric of area under the ROC (AUC of ROC).

We designed a paradigm to detect relevant events using gradually increasing thresholds to capture the gradual nature of attentional engagement. Figure 1 provides a visual explanation of our detection paradigm. Consider the EEG, EDA and IBI response traces that were recorded during the experiment. The timestamps of the data recordings can be separated in moments where interspersed stimuli were presented and where an event detection would thus be considered correct (True) and moments where no interspersed stimuli were presented and where an event detection would be considered incorrect (False). Rather than using the raw physiological responses, the detection paradigm is based on the PS between the participants as a function of time. Now let us define a threshold *t*. The moments in time where the synchrony is higher than *t* are marked as an event (Positive) and the moments in time where the synchrony is lower than *t* are marked as a non-event (Negative). Rather than using a single value for *t*, we consider a gradually changing threshold *t*, ranging from the minimum inter-subject correlation value to the maximum inter-subject correlation value. For each iteration of *t*, we can now define the true positives (TP), false positives (FP), true negatives (TN) and false negatives (FN). Using this, the true-positive rate or sensitivity (TPR) is then computed as,

$$TPR=\frac{TP}{TP+FN}$$

and the false-positive rate (FPR) or specificity as,

$$FPR=\frac{FP}{FP+TN}$$

Plotting TPR against FPR provides the receiver operating curve (ROC). Detection performance was assessed using the standard metric of the area under the ROC (AUC of ROC).

Chance level performance was assessed using permutations with randomized stimulus timing. In each permutation, the timing of all interspersed stimuli was randomized between the start and the end of the experiment. The same procedure as above was then applied to obtain the AUC of ROC metric of performance with random stimuli. This procedure was performed on 1000 renditions of such randomized data.

The above-mentioned procedure was repeated 2 × 3 × 4 times, namely for:

(1) Two attentional groups; considering PS between AA participants and PS between SA participants.
(2) Three stimulus types; considering as events (True) either blocks of beeps, emotional sounds, or both of these.
(3) Four physiological measures in which PS is computed; EEG, EDA, heart rate and a multimodal metric that is composed of PS in EEG, EDA and heart rate. To compose this multimodal metric, the PS in EEG, EDA and heart rate were each *z*-scored. The multimodal PS value at each timestamp was then computed as the average of the *z*-scored PS values in EEG, EDA and heart rate at that timestamp, for all timestamps ranging from zero to the end of the experiment.

In each condition, one-tailed two-sample t-tests were conducted to test whether detection performance was higher than chance level performance.

#### Correspondence Between Physiological Synchrony and Reported Engagement With the Audiobook

While for the SA group, the timing of short stimuli served as “ground truth” relevant events to compare to the moments of high PS, we did not know a priori what constituted relevant, engaging moments in the audiobook. To systematically examine whether moments of high PS were associated with moments of high relevance in the audiobook, we performed a follow-up test in which a second cohort of participants judged clips of the audiobook that were found to be associated with either high or low PS. We recruited 29 participants through the Prolific online experiment environment. All participants signed informed consent before participating. The participants received a small monetary reward for the invested time. We only included participants who indicated to be fluent in Dutch.

We selected clips based on continuous signals of PS among AA participants. We detected the positive and negative peaks in the signals using the ‘findpeaks’ function in MATLAB. For each measure (EEG, EDA, heart rate and the multimodal metric), the six peaks with highest positive peak-amplitude and six peaks with largest negative peak-amplitude were selected. For each detected peak, we created a 10 s sound clip, that was composed of the 10 s of audio before the detected peak. For four measures, this thus resulted in a total of 48 clips. Clips associated with peaks that were within 10 s of each other were considered to be overlapping, and were merged into one clip by using only the latest of the two clips in time. This resulted in a total of 38 clips that were presented to the participants.

The procedure of the online test was similar to the initial experiment. The participants were first presented with the same audiostream that was presented to the initial cohort of participants. The participants were instructed as participants from the AA group, i.e., to focus their attention on the narrative of the audiobook and ignore any interspersed stimuli as much as possible. After listening to the book, the participants were asked the same questions about the content of the narrative as participants in the initial cohort. We then presented the participants with the sound clips, each of them directly followed by a rating scale. Participants were instructed to rate the preceding clip using an 11-point Likert scale, ranging from 0 to 10. The lower the score, the more the participant’s experience corresponded to the words on the left side of the scale (Dutch: ‘verveeld’, ‘kalm’, ‘ontspannen’; Translated to English: ‘bored’, ‘calm’, ‘relaxed’). The higher the score, the more the participant’s experience corresponded to the words on the right side of the scale (Dutch: ‘geïnteresseerd’, ‘geboeid’, ‘emotioneel’, ‘intens’; Translated to English: ‘interested’, ‘fascinated’, ‘emotional’, ‘intense’). Using these words, we intended to capture mental states that are expected to be associated with perceiving relevant events, such as engagement, attention and arousal.

For each modality, we tested whether audio clips corresponding to a positive peak in PS were rated as more ‘engaging’ than audio clips corresponding to a negative peak in PS, using a Wilcoxon signed-rank test. Participants who answered less than three out of ten questions correctly on the questionnaire about the content of the audiobook were considered as not having participated seriously (AA participants in the main experiment answered 5.8 ± 2.0 questions correctly). This concerned three participants. Removing their data left us with data of 26 participants.

## Results

### Detection of Interspersed Stimuli Using Physiological Synchrony

**FIGURE 2:**
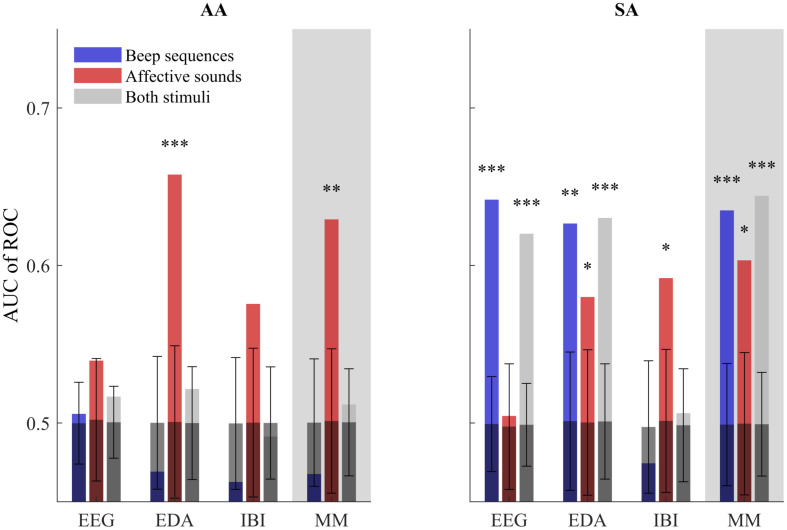
AUC of ROC metric of stimulus detection performance using PS in EEG, EDA, IBI and a multi-modal combination of the three (MM). Performance is shown for the AA and SA groups, when considering only beep blocks or emotional sounds as true positives and when considering both types of stimuli as true positives. In addition, the mean and standard deviation chance level detection performance based on 1,000 renditions with randomized stimulus timing is shown with test results comparing detection performance to chance level (^∗^*p* < 0.05,^∗∗^*p* < 0.01, ^∗∗∗^*p* < 0.001). Note that for the AA group, the emotional sounds but not the beeps are expected to draw (bottom-up) attention, i.e., for the AA group we expect high AUC for emotional sounds only. For the SA group, both beep sequences and emotional sounds are relevant and expected to draw attention.

Figure 2 and Table 1 show our measure of interspersed stimuli detection performance, the AUC of ROC as described in the methods. It is presented separately for AA and SA participants; in EEG, EDA, heart rate, and the multimodal metric; and for blocks of beeps, emotional sounds or both of these stimuli together as to-be identified events. Figure 2 and Table 1 also show the mean and standard deviation AUC of ROC of permutations with randomized event timing as a chance level baseline. Detection performance was largely in line with hypotheses 1 - 3. For the AA group, we found that, as expected, only the occurrence of emotional sounds could be predicted, using PS in EDA (*p* < 0.001) or the multimodal metric (*p* = 0.003). For the SA group, occurrences of beep blocks could be detected well above chance level by PS in EEG, EDA and the multimodal metric (*p* < 0.001, *p* = 0.002and*p* < 0.001, respectively). The occurrence of emotional sounds could be detected significantly better than chance using PS in EDA, heart rate and the multimodal combination (*p* = 0.043, *p* = 0.023,and*p* = 0.011, respectively). When stimuli were not differentiated according to stimulus type, detection performance was well above chance level for PS in EEG (*p* < 0.001), EDA (*p* < 0.001) and the multimodal metric (*p* < 0.001), but not for PS in heart rate.

**TABLE 1:**
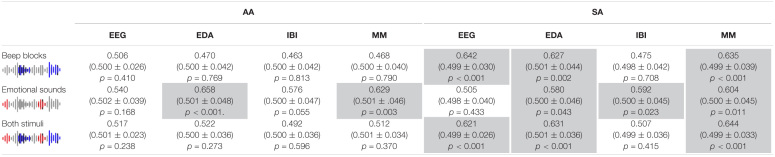
AUC of ROC metric of stimulus detection performance using PS in EEG, EDA, IBI and a multi-modal combination of the three (MM).

*Performance is shown for the AA and SA groups, when considering only beep blocks or emotional sounds as true positives and when considering both types of stimuli as true positives. In addition, the mean and standard deviation chance level detection performance based on 1,000 renditions with randomized stimulus timing is shown plus p-values of t-tests testing whether detection performance is significantly difference from chance level. Gray cells depict conditions in which detection performance is significantly higher than chance level (p < 0.05).*

### Correspondence Between Physiological Synchrony and Reported Engagement

**FIGURE 3:**
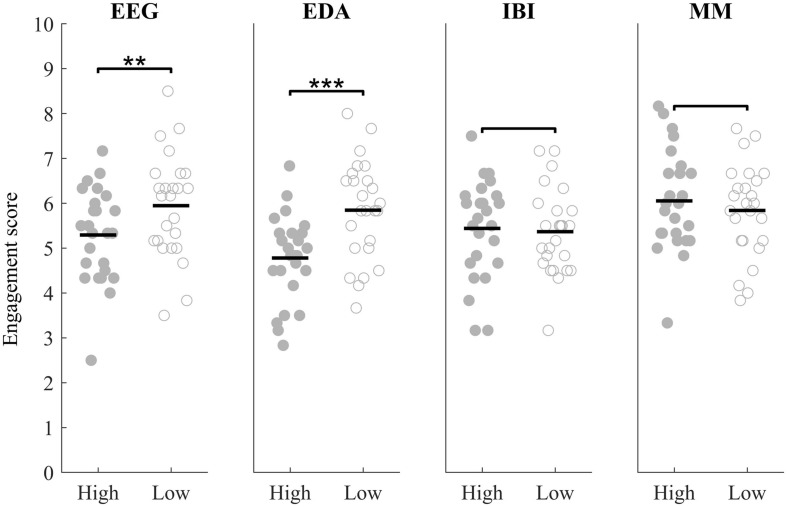
Self-reported engagement scores for audio clips corresponding to moments in the audiobook with high PS (closed markers) or low PS (open markers) in EEG, EDA, IBI and the multimodal metric (MM) (^∗∗^*p* < 0.01, ^∗∗∗^*p* < 0.001).

Figure 3 shows engagement ratings of audio clips corresponding to positive peaks and ratings of audio clips corresponding to negative peaks for PS in EEG, EDA, heart rate and the multimodal metric. Results did not follow our hypothesis that audio clips corresponding to positive peaks were rated as more engaging than audio clips corresponding to negative peaks. In fact, in EEG and EDA the opposite effect was found (Wilcoxon test statistic: *W* = −3.06, *p* = .002;*W* = −3.44, *p* < .001, respectively). In heart rate and the multimodal metric no significant difference between ratings corresponding to either positive or negative peaks was found (*W* = 0.70, *p* = 0.486, *W* = 0.87, *p* = 0.385).

## Discussion

In the sections below, the hypotheses as stated in the Introduction are discussed separately.

### Hypothesis 1: Attentional Instruction and Stimulus Type

We hypothesized that interspersed stimulus detection performance would depend on the attentional group (AA or SA) and the interspersed stimulus type (emotional sounds or beeps), due to bottom-up and top-down mechanisms of attention (Lang, 1995; Öhman et al., 2001; Schupp et al., 2003). For the SA group, we hypothesized that detection performance based on PS would be above chance for all interspersed stimuli, whereas for the AA group we hypothesized that detection performance would be above chance only for emotional sounds, but not for beeps. Results were largely in line with this hypothesis: for the AA group only the emotional sounds were detected with an accuracy above chance level, whereas for the SA group both stimulus types could be detected with above chance level accuracy. Note again that detection performance cannot be directly compared between the different stimulus conditions. There were differences in detection performance between the used physiological measures, these are discussed in the next section.

### Hypothesis 2: Physiological Measure and Stimulus Type

Besides the dependency of detection performance on the attentional group and interspersed stimulus type, we expected that detection performance would depend on the used physiological measure and stimulus type. As hypothesized, EEG worked best for the detection of blocks of beeps, while it did not work well for the detection of emotional sounds. We also found EDA to perform well for detecting blocks of beeps. For the detection of emotional sounds, we hypothesized that the peripheral measures (EDA and heart rate) would perform well relative to EEG. Indeed, for both attentional groups, PS in EDA and heart rate perform relatively well for the detection of emotional sounds. Detection performance was significantly above chance using EDA in both groups and using heart rate in the SA group, and near significance (*p* = 0.055) using heart rate in the AA group, whereas detection performance using PS in EEG was far from significant for emotional sounds in both groups. We think that the observed EEG PS differences between the types of stimuli are the result of both the difference in mental processing (top-down, effortful attention for beeps, versus bottom-up, affective processing for emotional sounds) and low level stimulus features. The beep blocks consisted of precisely-timed, repeated beep occurrences, with constant sound levels, while our emotional stimuli consisted of sounds with irregular sound profiles. The positive results obtained with peripheral measures provide further insight in the mechanisms underlying PS. Whereas previous findings on peripheral PS have been viewed in terms of social relation (Palumbo et al., 2017), we here show that PS in peripheral measures can also be explained by shared attentional engagement. It may be the case that shared attention also underlies results found in contexts of social relation.

### Hypothesis 3: Combining Physiological Measures

As the three used physiological measures vary with respect to their ability to reflect different mental states, we hypothesized that combining the physiological measures into a single multimodal metric of PS would result in relatively high detection performance when differences between stimulus types are disregarded. Indeed, the multimodal metric performs best when considering both emotional sounds and beeps as relevant events. Detection accuracies are slightly higher than for the best performing unimodal measure when considering emotional sounds or both types of stimuli but not when considering blocks of beeps. We expect that sensor fusion is not beneficial when variables are highly correlated (Hogervorst et al., 2014) – for example for physiological variables all reflecting mental effort – but that sensor fusion can benefit from tasks involving emotional processing besides effortful attentional processing. Besides potentially higher detection performance, the main advantage of multimodal PS seems to be the robustness regarding different types of stimuli, i.e., detection performance varies less between different types of stimuli than for single physiological metrics. In previous work similar effects have been found. For example, when using sensor fusion on machine learning models to distinguish between 13 emotional states, maximum performance was not higher for the multimodal metric, but performance was more robust across the range of emotional states (Verma and Tiwary, 2014). In the end, although adding sensors does not lead to much higher performance compared to the most suitable unimodal recording, a multimodal approach seems to enable detection of relevant events when it is unknown what the best measure for certain stimulus types is. Also note that it is not always known whether certain stimuli will induce mostly effortful cognitive or emotional processing; in many practical cases such processes can co-occur and vary between individuals.

### Hypothesis 4: Events in Audiobook

We hypothesized that audio clips corresponding to moments of highest PS would be post-hoc scored as more engaging than audio clips corresponding to moments of lowest PS, but our findings indicated rather the opposite. These findings may be caused by a mismatch between our index of PS and the rating scale of experienced engagement. Post-hoc qualitative analysis of the selected audio-clips revealed that part of the audio clips corresponding to very high PS in EEG coincided with short-term moments of tension or engagement, as expressed through keywords (e.g., swear words) and salient intonation (e.g., a phrase spoken in a very indignant manner). This is in line with earlier work, where moments of high PS in EEG were found to correspond to moments in video clips marked by a high level of short-term suspense, tension or surprise, such as the sight of a gun (Poulsen et al., 2017). Indeed, emotional images and sounds that are rated as highly arousing induce responses in peripheral and central physiological measures (Bradley and Lang, 2000; Lang and Bradley, 2007), which in term may lead to strong PS. In our used audiobook, the keywords that may have driven the particularly high PS contained relatively little important information about the narrative of the story. It seems that this could have been the aspect rated by participants using our engagement scale, leading to a mismatch between self-reported engagement and PS. However, this speculation would need to be investigated further, preferably without having to rely on varying engagement judgements after the fact, but for instance with systematic sentiment analysis (Wöllmer et al., 2013). It is important to further specify what types of attentional engagement can and cannot be captured by PS and how that is dependent on the psychological measure used. Attentional engagement to well-timed events will be better reflected in PS than attentional engagement to less well-timed event on a more abstract level.

### Limitations

It should be noted that the stimulus detection performance when not taking stimulus type into consideration (‘both stimuli’) were mainly driven by detection performance of beep blocks. These beep blocks were interspersed for a total of 810 s, whereas the emotional sounds were only interspersed for a total of 216 s. The stimuli also differed on other aspects. For instance, the beep blocks consisted of precisely timed beeps with immediate stimulus onset equal across trials, whereas the emotional sounds all differed in sound profile. For the AUC of ROC metrics when considering detection of both types of stimuli, beep blocks thus influence the performance metric more than the emotional sounds. While this can be seen as a limitation, this is exemplary for real life situations, where one is interested in detecting relevant, attentionally engaging events, without further specifying or knowing the different types of stimuli, and the proportion of in which they occur; i.e., in such a situation, the ‘both stimuli’ situation is the default.

We must also note that our simple multimodal approach is certainly not the optimal approach to combine data. In particular, we expect that detection performance can be enhanced by compensating for differences in response latencies across measures. To illustrate the difference in response latency, in response to the same set of emotional sounds, response peak latency ranges from a few 100 ms for EEG to multiple seconds in EDA heart rate (Bradley and Lang, 2000; Hettich et al., 2016). In this paper we simply averaged over response traces in a point-wise fashion, meaning that response-induced peaks may be spread out and their amplitude reduced. Our current results should therefore be interpreted as a first confirmation that multimodal sensor fusion can be of added value, but we expect that other approaches can greatly enhance performance. In future work we would like to explore other methods for the combination of physiological measures into a multimodal metric of PS.

## Conclusion

We determined PS in EEG and EDA, heart rate and a multimodal fusion of these three sensors in two groups of participants, that were instructed to attend either to the narrative of an audiobook or to interspersed auditory events. We found that PS could detect the relevant interspersed stimuli with accuracies well above chance level, but also found that moments in the audiobook corresponding to high PS were not rated as more engaging than moments corresponding to low PS. Our results support the notion that PS can be valuable when interested in the course of attentional engagement over time. Currently the relation between PS and engagement is only established for well-defined, interspersed emotional or effortful cognitive stimuli, whereas the relation between PS and a more abstract self-reported metric of engagement is not yet established. We further note that obtained results vary between the used physiological measures. Interesting from a user perspective, EDA worked best overall. These results should enable researchers to monitor PS in situations where intrusive EEG measurements are not suited. However, we also note that the optimal physiological metric may be dependent on the goal of a study and suggest to choose a measure matching with the stimulus of interest. EEG works especially well for well-timed effortful cognitive stimuli, heart rate works especially well for emotional stimuli and EDA works quite well on both types of stimuli. When the stimulus type is unknown, a multimodal metric may work best as it seems most robust across a broad range of stimuli.

## Data Availability Statement

The data presented in this study can be found on the Open Science Framework: https://osf.io/8kh36/. The code can be obtained from the corresponding author upon request.

## Ethics Statement

The studies involving human participants were reviewed and approved by the TNO Institutional Review Board. The participants provided their written informed consent to participate in this study.

## Author Contributions

A-MB and IS conceived and designed the study. IS and NT performed the experiment. IS analyzed the data with input from A-MB, JE, and NT. IS wrote the first draft of the manuscript. All authors thoroughly reviewed and revised the manuscript drafts and approved the submitted version.

## Conflict of Interest

The authors declare that the research was conducted in the absence of any commercial or financial relationships that could be construed as a potential conflict of interest.
